# Inferring a Transcriptional Regulatory Network from Gene Expression Data Using Nonlinear Manifold Embedding

**DOI:** 10.1371/journal.pone.0021969

**Published:** 2011-08-12

**Authors:** Hossein Zare, Mostafa Kaveh, Arkady Khodursky

**Affiliations:** 1 National Institutes of Health, Bethesda, Maryland, United States of America; 2 Department of Electrical and Computer Engineering, University of Minnesota, Minneapolis, Minnesota, United States of America; 3 Department of Biochemistry, Biophysics and Molecular Biology, University of Minnesota, St. Paul, Minnesota, United States of America; University of Glasgow, United Kingdom

## Abstract

Transcriptional networks consist of multiple regulatory layers corresponding to the activity of global regulators, specialized repressors and activators as well as proteins and enzymes shaping the DNA template. Such intrinsic complexity makes uncovering connections difficult and it calls for corresponding methodologies, which are adapted to the available data. Here we present a new computational method that predicts interactions between transcription factors and target genes using compendia of microarray gene expression data and documented interactions between genes and transcription factors. The proposed method, called Kernel Embedding of Regulatory Networks (KEREN), is based on the concept of gene-regulon association, and captures hidden geometric patterns of the network via manifold embedding. We applied KEREN to reconstruct transcription regulatory interactions on a genome-wide scale in the model bacteria Escherichia coli (E. coli). Application of the method not only yielded accurate predictions of verifiable interactions, which outperformed on certain metrics comparable methodologies, but also demonstrated the utility of a geometric approach in the analysis of high-dimensional biological data. We also described possible applications of kernel embedding techniques to other function and network discovery algorithms.

## Introduction

Expression of a living genome is a result of interactions between transcriptional regulators and their target sequences. A set of direct interactions, or primary connectivity, for each regulator is known as a regulon. A complete set of cellular regulons and connectivity among them make up for a transcriptional regulatory network. Transcriptional connectivity are affected by a multitude of factors, including but not limited to: i) activity of proteins associated with chromosomal DNA (nucleoid associated proteins in bacteria and histones in eukaria); ii) activity of transcriptional activators and repressors; iii) accessibility of target DNA; iv) composition and activity of RNA polymerase; v) level of DNA superhelicity; vi) metabolic state of the cell. Each of these factors can be viewed as one dimension in the intrinsically high-dimensional regulatory space.

Geometrically, a transcriptional network can be viewed as a graph in a metric space where the probability of connection between two nodes (genes) depends on their distance, i.e., similarity in transcriptional profiles across multiple dimensions of the regulatory space. Such representation assures that the genes of any one regulon are located in greater proximity to each other than the genes belonging to different regulatory units. Thus the data points corresponding to individual members of a regulon will be constrained in a well defined sub-space, indicating that a topological object, also referred to as a manifold, underlies transcriptional data characterizing any given set of co-regulated genes. On a manifold of gene expression, similar expression profiles are points in the local neighborhood of the manifold. Since, by definition, any manifold is locally Euclidian, i.e. nearly two-dimensional on a local scale, a transcriptional manifold can be naturally learned by techniques that explore this manifold's property. Such approach should result in the reconstruction of a nearly complete regulatory network as an ensemble of locally learned regulatory neighborhoods.

In recent years, many innovative approaches have been put forward in order to solve the task of reconstruction of regulatory transcriptional networks on various scales, either only from expression data or from a combination of expression data with transcription factor binding and/or sequence information. The plethora of learning philosophies and computational styles can be represented by Bayesian networks [1]–[3], relevance networks [4]–[6], module-inferring algorithms [7]–[9], as well as techniques based on matrix decomposition [10], [11]. Despite their application successes, these and other approaches circumvent rather than adapt to the intrinsic properties of data and implicit nature of the networks. In fact, high-dimensionality and non-linearity of the transcriptional regulatory space along with the pervasiveness of hidden network connections can be more naturally accounted for by a geometric approach. Such approach can be based on manifold learning [12]–[14] and kernel embedding [15], [16], which provide a mathematical framework not only for nonlinear dimensionality reduction in the data, but also for capturing the structure and geometric distribution of the data. These methods have already been successfully applied to high-dimensional data such as images and motion pictures. They have also been applied to several problems in bioinformatics. Kernel approaches were proposed in [17], [18] as a means of integrating different sources of data for clustering and classification purposes. More recently, functional distances between Gene Ontology (GO) annotations have been defined by means of a diffusion-type manifold embedding in order to uncover relationships between protein domains' structure and function [19].

In this study we aimed to provide a framework for the application of the nonlinear manifold learning techniques to reconstruct transcription regulatory networks. For this purpose, we considered a transcription regulatory network as a network which comprises of several connected components (regulons) with a structure different from that of a random network. We referred to this specific structure of the network as a geometric connectivity pattern. We assumed that gene expression of the members of different regulons are sampled from different manifolds which can explain the network connectivity pattern, and usd kernel embedding reduction methods to simultaneously capture the nonlinear correlation and the underlying connectivity pattern in the gene expression data. We defined the regulon-based association scores between genes and a transcription factor's core regulon to combine uncovered hidden connectivity patterns in gene expression data with known connectivity patterns in order to reconstruct a transcription regulatory network of E. coli.

## Methods

### Gene-Regulon Association Score

In traditional network reconstruction methods, the associations between genes and regulators have been based on different distance or similarity measures between the target genes and the genes coding for transcription regulators. However, a more meaningful association can be made by defining a similarity between the target genes' expression profiles and activity profiles of the regulators. Direct measurement of the activity profiles of regulators, if at all possible, would require a complicated biological experimental setup. Nonetheless, the relevant information regarding activity profiles of a regulator can be captured from the expression profiles of the members of its core regulon [10], [20], and this is the main motivation behind defining a gene-regulon association score.

The advantage of a gene-regulon score can be explained in the context of mutual information theory. Transcriptional regulatory networks can be analyzed by computing the mutual information between mRNA abundance profiles of genes and their regulators [5], [6]. However, because the activity profile of a transcription factor, which determines its propensity to regulate target genes, may not be similar to its mRNA profile, it is more appropriate to define mutual information between transcriptional activities of individual genes and regulons to which they belong. Let genes *x_1_, x_2_, …, x_n_* co-regulated by the same transcription factor form the regulon set Ω. Then the activity profile of the transcription factor denoted by latent variable *Z*, which explains this regulation, minimizes the conditional mutual information between the gene variables and is given by [22]:
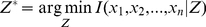
(1)Given a regulon, one can estimate *Z*, Z^*^, which in many cases is different from the gene expression profile of the transcription factor. Therefore, a more accurate prediction of gene regulatory interactions can be achieved by assigning genes according to transcription factors' activity profiles, the Z^*^'s. Since the estimation of *Z* is not an easy task and requires very large amount of data, the dependency between genes and their regulators can be approximated by defining the gene-regulon association score as follows.
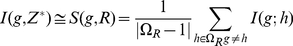
(2)where I(g, Z^*^) is the mutual information between gene *g* expression profile and the estimated activity profile of its regulator, Z^*^, *g* and *h* are genes, *R* is a regulon and Ω*_R_* is a set containing members of the regulon. Then the true regulator for a gene *g* maximizes *S(g, R)* among all regulators. The mutual information captures the nonlinear dependency in the data but it cannot capture the geometric connectivity patterns. Having conceptually shown that the gene-regulon score is a better measure of association between genes and transcription factors, in the following we propose a new association measure which simultaneously captures nonlinear dependencies and geometric connectivity patterns in the gene expression data.

### Capturing connectivity patterns in transcriptional regulation through kernel embedding

Given gene expression data across many diverse conditions, it is reasonable to assume that each regulon is only responsive to a subset of conditions. Therefore, the intuitive and natural approach is to identify the manifold for each regulon where all the data points belonging to the regulon lie. Then, one can define a distance between genes and the manifold for each regulon. However, instead of explicitly learning the manifold from the data, an alternative approach would be to use kernel embedding matrices, which preserve local similarity, to measure the association between genes and regulons.

Given a transcription factor and its core regulon we define the association score between genes and the regulon as:
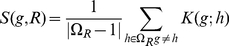
(3)where Ω*_R_* is a core regulon for the regulon *R* and | Ω*_R_* | is the cardinality of *R*, and *K* is a kernel embedding matrix. The choice of the kernel embedding method depends on how well it can capture the geometric connectivity pattern in the data. The locally linear embedding (LLE) algorithm presented in [12] is a nonlinear dimensionality reduction algorithm, which recovers global nonlinear structure through locally linear fits. It first reconstructs each data point in the original space from its neighbors and assumes the same reconstruction coefficients are valid in the embedding space. Let *W* be a reconstruction weight matrix in LLE originally introduced in [16], or a normalized local similarity matrix which has been used in [19], whose *i*-th row sums to unity. Then a LLE kernel matrix can be defined as follows [16]. Let *e* be a uniform and unity vector of size *N* (its elements are *1/√N*), and set:
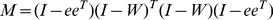
(4)Then an LLE kernel can be formed by the following:
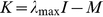
(5)where λ_max_ is the largest eigenvalue of *M*. Other forms of the kernel embedding matrices such as ISOMAP kernel, Laplacian kernels [16] and the diffusion kernel of powers can also be defined [15]. However, we observed that the LLE kernel from the local similarity matrix constructed using correlations produced better results.

## Results

Genome-wide expression data and information about known interactions between genes and transcription factors have been used to predict regulatory targets. We extracted core regulon information for each TF from the RegulonDB database [24]. The most recent data set contains known interactions for 137 transcription factors with at least 3 interactions. The complete set covers 1446 genes with a total of 3213 interactions. We also used two different microarray gene expression data sets. Our first set is microarray gene expression data for more than 100 arrays representing 46 biologically distinct conditions. These conditions covered a wide spectrum of environmental and genetic perturbations. The environmental perturbations, in addition to those described in [23], included different amino acid and nucleotide additions and limitations [20]. The second data set used in our study has been published in [5] and was obtained from Many Microbe Microarray database (M^3D^; http://m3d.bu.edu). This set contained expression levels of E. coli genes across 524 arrays corresponding to 189 different experimental conditions. It should be noted here that not only these two data sets covered very different genetic or environmental perturbations, but they were also collected on two different microarray platforms: cDNA microarrays and Affymatrix Genechips.

As outlined in the method section, the proposed algorithm involves two steps. On the first step, the gene expression data is used to construct the kernel matrix. At this stage, there is no need for any regulon information. On the second step, the association score between a gene and a regulon is calculated. At this stage, the knowledge of the regulon is used. However, according to equations 2 and 3, if a gene is part of a regulon it will be excluded from the calculation of the association score between the gene and the regulon. Therefore, the exact leave-one-out cross validation procedure is inherent in the prediction process. To construct kernel matrices, we first constructed the Pearson linear correlation matrix and pairwise mutual information matrix for each data set. We computed pairwise mutual information based on a B-spline smoothing and discretization method, as in [21], using Matlab program provided in [5]. Then, the local similarity matrices were constructed from these matrices by keeping only the K nearest neighbors of each gene and putting other elements to zero, while forcing the matrix to remain symmetrical. This guarantees that the resulting matrix has the properties of a similarity matrix. We compared the prediction accuracy of the proposed method (KEREN) with a Gene-TF method (relevance network) and two other Gene-Regulon based approaches using either mutual information or correlation matrices. We used the set of known interactions and compared different approaches by their ability to recover these interactions. We assumed that each method should assign at least one regulator to each gene with known interactions. The assignment of the regulators to genes was made by the following rules. Each method provides the association score between the genes and regulators. For example, in the case of relevance network (CLR network [5]), the scores are CLR scores between genes and TFs. In the regulon based approaches, the association scores are the similarity scores between genes and core regulons. For each gene, we ranked regulators based on their association scores with that gene. A regulator that had the maximum association score with the gene was assigned to that gene. A second regulator was assigned to the gene if the corresponding association score was greater than the average of the association scores of the genes assigned to that regulator in the first round. This procedure was repeated and assignments were made, if warranted by the association score, for lower ranking regulators as well. We refer to the number of rounds of assignments as *P*.

The recall and precision measures were used to compare different approaches. We define recall as a fraction of genes with known interactions for which at least one interaction was recovered. We define precision as a fraction of predicted interactions which were among known interactions. The reason for using a fraction of genes instead of a fraction of all interactions in calculating recall is that we do not expect that all of the interactions for a gene are being realized in a particular data set. However, we expect that at least one of the known interactions for each gene can be explained by an expression data set which covers reasonably large number of experimental conditions. Nonetheless, the values of recall defined as the fraction of all known interactions recovered correctly were not significantly different from those reported here.

**Figure 1:**
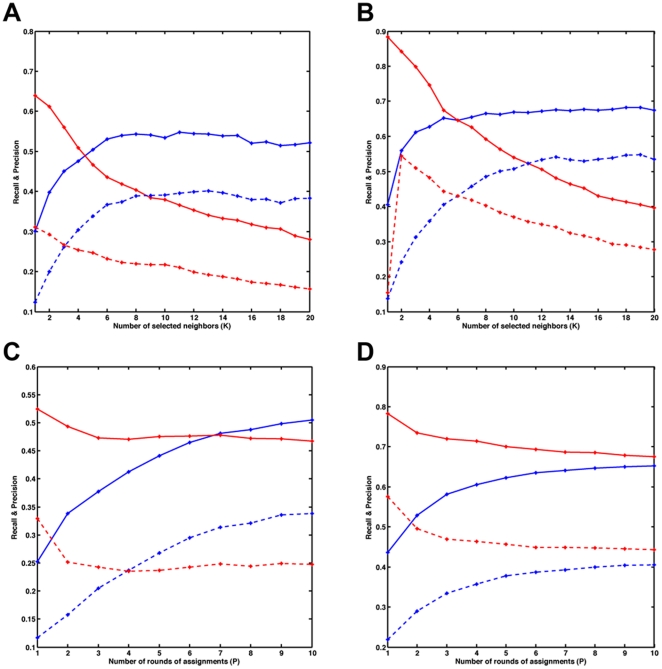
Performance of KEREN on two data sets of different size. (A) Comparison of recall (blue line) and precision (red line) for cDNA microarray data set versus K, the initial number of selected neighbors, for fixed value of P = 10. (B) the same as (A) for Affymatrix data set. (C & D) An effect of the proposed assignment procedure (parameter P) for the cDNA and Affymatrix data set, respectively. K is equal to 5 in this case. Dashed lines correspond to the performance when operons are accounted for.

Figure 1 (A & B) depicts recall (blue line) and precision (red line) values with respect to the values of *K*, for a fixed parameter *P = 10*, for two different data sets. Since in E. coli some genes are organized in operons and are co-transcribed, we also plotted recall and precision (dashed-lines) that accounted for the operon structure. To account for the bias that could be introduced by operonal organization of genes on the chromosome, we made the following adjustment. If a gene belongs to a regulon and also is part of an operon, the whole operon was excluded from the regulon when calculating the gene-regulon association score. As it can be seen from the figures, in both cases, the detrimental effect of increasing *K* is more pronounced in precision than in recall, which is indicative of increasing the number of predictions with no or few additional predictions belonging to the set of known interactions. Figure 1 (C & D) shows the effect of the assignment procedure (parameter *P*) on recall and precision for a fixed number of nearest neighbors *K = 5*. Again, we can see that the proposed assignment procedure is very robust and has a positive impact on recovering interactions.

Table 1 shows a comparison between different approaches for the two data sets. A gene-TF based approach is a CLR version of the relevance network using mutual information. However, unlike in the original work [5], which used the threshold and therefore gave limited number of predictions, we required all algorithms to provide at least one prediction for each gene with known interactions. We also present four different versions of the gene-regulon based approach. These versions are different in how they compute the similarity between genes and regulons. The first version used the correlation values, the second - pairwise mutual information values, the third and forth were KEREN method and used LLE kernel embedding matrices constructed from mutual information or correlation matrices, respectively. As it can be seen from the table and figures, the second data set provided more accurate predictions, presumably because it sampled a greater number of dimensions of the regulatory space by covering many more experimental conditions. However, for both data sets across different approaches, the gene-regulon based approach using LLE kernel yielded the most accurate predictions. Interestingly, the LLE kernel constructed from the correlation matrix performed better than the LLE kernel constructed from mutual information. We argue that LLE kernel by itself can capture the nonlinear correlations and therefore the property of mutual information to capture nonlinear dependencies does not provide additional value in this case. However, because of the loss of information due to discretization of the data, the estimation of mutual information is more sensitive to a sample size than the estimation of correlation. Therefore, the kernel matrix constructed from the mutual information matrix is weaker than the kernel matrix constructed from the correlation matrix. This is more apparent when one compares the performance of the kernels for the data set with a smaller number of samples. Therefore, we note that the reason for a better performance of a kernel derived from correlations is that the estimation of the correlation from the data was more accurate than the estimation of the mutual information from the data and this resulted in a more accurate local similarity matrix, which in turn affected the constructed kernel. The performance of the kernel approaches were obtained using the *K = 5*, number of neighbors and *P = 10*, number of rounds of assignments.

**Table 1:** Performance Comparison.

	cDNA Data Set		Affymatrix data set	
Method/Algorithm	Recall	Precision	Recall	Precision
A	13.5	9.5	26.8	17.1
B	31	10	42.6	14.5
C	30	13.6	47.2	25
D	44.7	40	62.3	63.2
E	50.5	46.7	66	68

Recall and precision values are in % for two microarray data sets. Methods/Algorithms are: (A) Gene-TF, relevance network, (B) Gene-Regulon using a correlation matrix, (C) Gene-Regulon using a mutual information matrix, (D) KEREN, Gene-Regulon using an LLE kernel matrix derived from a mutual information matrix, (E) KEREN, Gene-Regulon using an LLE kernel matrix derived from a correlation matrix.

Since the KEREN method takes advantage of known interactions, we compared its performance with that of a recently published supervised learning algorithm (SIRENE) [25]. To be fair in our comparison we applied KEREN (with *K = 5* and *P = 10*) to the same gene expression data set and the same interaction data set used in SIRENE paper [25]. In Table 2, we reported the precision values for different values of recall for KEREN and SIRENE with and without account for annotated operons. SIRENE is a supervised algorithm and uses a kernel function for pairs of genes to learn a support vector machine (SVM) classifier for each TF. Our algorithm based on kernel embedding outperforms SIRENE because it is capable of capturing connectivity patterns in the data. This results from the fact that, unlike the kernel function used in SIRENE, the embedding kernel similarity between two genes is influenced by all genes and especially by their neighbors.

**Table 2:** Comparison of KEREN with SIRENE.

Method/Algorithm	Recall = 80%	Recall = 75%	Recall = 70%	Recall = 65%	Recall = 60%	Recall = 50%
KEREN	30	38	44	47	50	54
SIRENE	16	18	23	29	35	50
KEREN-bias	65	75	82	86	88	91
SIRENE-bias	62	70	75	82	86	90

Comparison between precisions (%) of KEREN and SIRENE (with operon structure accounted for in the first two rows and not – ‘bias’) at different levels of recall. The values for SIRENE were taken from [25].

**Figure 2:**
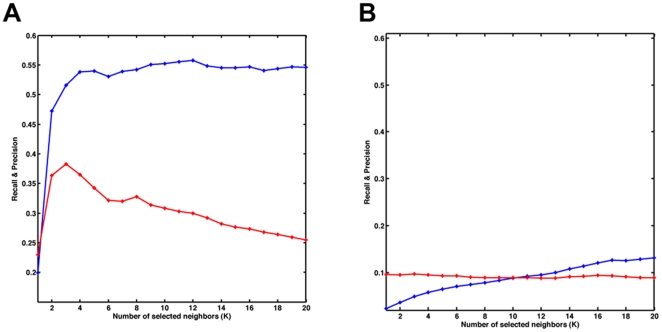
Performance of KEREN using Laplacian kernel. (A) Comparison of recall (blue line) and precision (red line) for KEREN when Laplacian kernel instead of LLE is derived from the correlation matrix of the Affymatrix data set. (B) Comparison of recall and precision for the Affymatrix data when LLE kernel is constructed from correlation matrix of randomized data.

As an alternative to LLE kernel embedding technique, figure 2A depicts the recall and precision for Affymetrix data set when Laplacian kernel is used in KEREN method with *P = 10*. The Laplacian kernel [14], [16] can be formed from the local similarity matrix W, first by forming the graph Laplacian, *L = D-W*, where *D* is a diagonal matrix called degree matrix. The diagonal elements of *D* are sum of the row elements of W. Then the Laplacian kernel is defined by taking pseudo-inverse of graph Laplacian. Although the LLE kernel performance is better than that of Laplacian, the Laplacian shows slightly more robust behavior with respect to the number of neighbors, which is due to its property to capture both short and longer range interactions on the graph. However, these longer range interactions derived from local similarity matrix may not be true interactions, and in turn may affect the overall performance of the Laplacian kernel.

We also carried out the following procedure to determine a false discovery rate (FDR) of our prediction procedure. We permuted the gene expression data for each gene across experimental conditions and applied the algorithm to the permuted data. The randomization process and the simulation were repeated 100 times. We only applied this procedure to the data set with a larger sample size and used the LLE kernel constructed from the correlation matrix. We calculated the recall and precision as defined above. Figure 2B shows the recall-precision performance for different values of *K* for randomized data. Both recall and precision for randomized data remained significantly low for all values of K. To estimate the FDR, one can calculate the true positive (TP) value when applying algorithm to real data and the false positive (FP) value when applying the algorithm to randomized data. Then one can approximate the false discovery rate as *FDR = FP/TP*. To be more precise, considering that the total number of predictions in two cases might be significantly different, we defined *FDR = Precision_R_/Precision*, where Precision is a precision value when using real data, and Precision_R_ is a precision value when using randomized data. This resulted in the FDR value of less than 10% when averaging over 100 randomized data sets.

## Discussion

We have presented a computational method based on kernel embedding for predicting interactions between transcription factors and their gene targets. Our method captures network geometric patterns and incorporates them to expand known regulons. We have shown the power of the embedding to accurately reconstruct gene regulatory networks, and below we discuss other possible applications of this methodology. Over the course of the past few years, several approaches have been presented in the literature to integrate gene expression data with other types of genome-wide data to identify targets of transcription factors and to discover network modules. GRAM [7], ReMODiscovery [9] and COGRIM [8], to name a few, are among those which combine gene expression data, ChIP-chip and motif data to discover regulatory modules. For example, ReMoDiscovery algorithm first detects large modules which contain tightly co-expressed genes sharing common regulators and motifs, and then extends the modules by computing mean seed profile and assigns the remaining genes to modules based on their similarity to seed profiles. On the other hand, GRAM algorithm detects core modules for a subset of regulators based on binding data and then extends the modules by adding genes which are at certain distances from the gene expression center of modules, and at the same time satisfy a relaxed p-value threshold for their binding affinity to modules' regulators. While these algorithms have their own advantages and shortcomings, which is not the focus of our discussion here, they both compute the similarity between genes and modules in the expression domain using linear correlations. We saw that linear correlations only capture a fraction of the information in the data and, therefore, the accuracy and performance of these algorithms are limited. However, one can expect a big boost in the performance of these algorithms by using the kernel embedding matrices. This is due to the property of the kernel matrices in reducing the noise and also capturing the connectivity patterns in data.

In a more recent work [26], with motivation similar to the current work, an attempt has been made to complete the transcriptional regulatory network of E. coli using gene expression data, motif data and the knowledge of known interactions. A semi-supervised algorithm was presented to accomplish the task. The method used information about known interactions and the gene expression data to learn multi-logistic regression classifiers and rank genes as regulated by a transcription factor based on their probability of belonging to each class. For each TF, 3 classifiers were learned from the data, which in turn requires the estimation of 2*M logistic regression coefficients from the training data. However, when *M* (the number of experimental conditions) is large and the number of training samples is low (which is the case for many TFs) the estimation of too many parameters is unsound and would result in classifiers with low predictive power due to over fitting. On the other hand, many genes respond to a limited number of conditions and expression values across many other conditions should account for noise. An alternative approach to address this issue is to use kernel embedding matrices to embed the data in a low dimensional domain. The coordinates of data on the embedded domain are the eigenvectors corresponding to the *N* largest eigenvalues of the kernel matrix. The data in the embedded space not only has smaller dimensionality, but also captures the connectivity patterns in gene expression data. One then can use the embedded data instead of original gene expression data in logistic classifier in [26]. This procedure would increase the prediction accuracy in two different ways. First, the features in data will be less noisy and more informative and second, the number of parameters to be estimated for logistic regression problems will become much smaller, 2*N instead of 2*M where *N<<M*.

### Conclusion

Genome-wide transcriptome and interactome data allow for systematic discovery of interactions between transcriptional regulators and their targets. Accurate prediction of such interactions, which is essential for understanding phenotypic outcomes of genetic and environmental perturbations, depends on the quality of models capturing essential regulatory features and on their underlying assumptions. Geometric connectivity patterns among the members of the regulon is one of the features hidden in the gene expression data that, when properly captured, can greatly enhance the prediction accuracy of the network reconstruction methods. We introduced an effective approach to predict interactions between regulators and their target genes using kernel embedding matrices called Kernel Embedding of Regulatory Network (KEREN). KEREN uses kernel embedding of gene expression data to simultaneously capture nonlinear dependencies and geometric connectivity patterns in the data. Then, it takes advantage of available interactome data to discover new interactions between genes and regulators using a gene-regulon based association method.

We demonstrated that the application of the kernel embedding matrices in combination with gene-regulon based association strategy results in more reliable identification of many known as well as previously uncharacterized regulatory interactions. We should mention that the power of a regulon-based association strategy and of any supervised or semi-supervised inference methods relies on the availability of the interactome data, and without such information these supervised algorithms are not applicable. However, with the help of ChIP-chip and ChIP-sequencing technology, it should be possible to obtain sufficient amount of interactome data de novo to satisfy the connectivity matrix requirements. We also discussed how several previously known and established biological discovery algorithms can benefit from kernel embedding matrices constructed from gene expression matrices. Finally we would like to emphasize an important application of kernel embedding matrices to the analysis of gene expression data sets derived from different sources and/or platforms. Whereas combining such data sets directly may not be feasible, the kernel matrices from each data set can be easily combined in an algebraic manner and the resulting matrix can be treated as a single kernel matrix in any discovery algorithm.
